# Seedborne Pathogenic Fungi in Common Bean (*Phaseolus vulgaris* cv. INTA Rojo) in Nicaragua

**DOI:** 10.1371/journal.pone.0168662

**Published:** 2016-12-20

**Authors:** Delfia Marcenaro, Jari P. T. Valkonen

**Affiliations:** 1 Nicaraguan Institute of Agricultural Technology (CNIAB-INTA), Managua, Nicaragua; 2 Department of Agricultural Sciences, University of Helsinki, (Latokartanonkaari 7), Helsinki, Finland; Korea University, REPUBLIC OF KOREA

## Abstract

Common bean (*Phaseolus vulgaris* L.) is an important legume with high nutritional value. In Nicaragua, certified healthy seeds of local bean varieties are not available, and seedborne fungi have gained little attention. Here, were surveyed seedborne pathogenic fungi in an important local bean cultivar, ‘INTA Rojo’. Beans grown in the four main production areas in Nicaragua (Boaco, Carazo, Estelí, Matagalpa) for future use as seed stock were sampled from four seed storehouses and six seed lots. A total of 133 fungal strains were isolated from surface-sterilized beans and inoculated to healthy lima beans (*Phaseolus lunatus*) under controlled conditions. Eighty-seven isolates caused symptoms of varying severity in the seedlings, including discoloration, necrotic lesions, cankers, rot, and lethal necrosis. Pathogenic isolates were divided into eight phenotypically distinguishable groups based on morphology and growth characteristics on artificial growth medium, and further identified by analysis of the internal transcribed spacer sequences (ITS1 and ITS2) of the ribosomal RNA genes. The pathogenic isolates belonged to eight genera. *Fusarium* spp. (*F*. *chlamydosporum*, *F*. *equiseti*, *F*. *incarnatum*), *Lasiodiplodia theobromae*, *Macrophomina phaseolina*, and *Penicillium citrinum* were the most damaging and common fungi found in the seed lots. Furthermore, *Corynespora cassiicola*, *Colletotrichum capsisi*, *Colletotrichum gloeosporioides*, *Aspergillus flavus*, and *Diaporthe* sp. (*Phomopsis*) were seedborne in cultivar ‘INTA Rojo’ and found to be pathogenic to bean seedlings. This study reveals, for the first time, many seedborne pathogenic fungi in beans in Nicaragua; furthermore, prior to this study, little information was available concerning *F*. *equiseti*, *F*. *incarnatum*, *L*. *theobromae*, *C*. *cassiicola*, and *Diaporthe* spp. as seedborne pathogens of common bean. Our results lay the basis for developing diagnostic tools for seed health inspection and for further study of the epidemiology, ecology, and control of the pathogenic fungi of common beans in the field.

## Introduction

The common bean (*Phaseolus vulgaris* L.) is an important grain legume that is widely grown, especially in Latin America and Africa [1]. It has high nutritional value owing to its notable content of protein, vitamins, zinc, iron, and fiber [2,3]. In Nicaragua, common bean and maize (*Zea mays* L.) represent the main crops for income generation and food security [1,4], and there is an emphasis on breeding bean cultivars that are better adapted to local growth conditions. Advanced locally selected cultivars such as ‘INTA Rojo’ and ‘INTA Cardenas’ are prioritized by the Nicaraguan government for large-scale production in the cropping systems used by small-scale farmers. ‘INTA Rojo’ was bred in Zamorano School, Honduras, by crossing the cultivar (cv.) ‘INTA Canela’ with cv. ‘DICTA 105’. It is one of the most important bean cultivars in Nicaragua owing to its high yield, drought tolerance, adaptability to different environmental conditions, red skin (preferred by local consumers), good flavor, and short cooking time [5,6].

In Nicaragua, common bean is mainly produced carried out on small farms with limited access to advanced agrotechnology and fertilizers. A severe shortcoming is the lack of healthy seeds because the greatest yield losses owing to pathogens occur when seeds used for planting are infected. Seedborne pathogenic fungi can prevent germination, kill seedlings, or reduce plant growth by damaging the roots and vascular system, which prevents the transport of water and nutrients [7,8]. Seedborne pathogenic fungi that cause losses of yield and quality of common bean worldwide include, but are not limited to, *Macrophomina phaseolina* (Tassi) Goid., *Fusarium oxysporum* (Schltdl.) Fr., *F*. *solani* (Mart.) Sacc., and *Rhizoctonia solani* Kühn [9,10].

Production of healthy, certified seed beans for local use is an important goal in Nicaragua. Although information exists concerning pathogenic fungi in many crops in Nicaragua, little knowledge is available concerning those of common bean [11]. Hence, knowledge of the locally prevailing seedborne pathogenic fungi in bean needs to be improved so pertinent seed inspection procedures may be carried out. Therefore, the aim of this study was to identify fungi transmitted in the beans (‘INTA Rojo’) and to test their pathogenicity on seedlings.

## Materials and Methods

### Analysis of emergence and symptoms of seedlings

Beans inspected for seedborne fungi were harvested from Boaco, Carazo, Estelí, and Matagalpa, representing the four main bean growing areas in Nicaragua. The crops were grown during the “primera” season (May–August, 2008) of the year. Samples from six storehouses were taken in August–October. The storehouses were owned by cooperatives established by small holders. Each storehouse contained 8–15 t of beans harvested from 10–20 farms. Guidelines of the International Seed Testing Association [12] were followed in taking six subsamples from stored beans of a storehouse, combining them (final sample size 1.5–2.0 kg/storehouse), and blending to homogeneity.

For testing emergence, eight subsamples (50 beans each) were taken from each of the six samples. Each subsample was planted in a separate tray (38 x 24 cm, depth 19 cm) filled with sterilized growth medium (autoclaved at 121°C for 2 h) consisting of washed sand and peat. The trays were organized according to a completely randomized design in a growth room (20–22°C) in dim light (photoperiod 11 h). Emergence of seedlings was observed for 15 days, after which all plants were gently removed from soil, rinsed with water, and observed for disease-like symptoms in the stem base and roots. One-way analysis of variance and comparison of means based on the Tukey test (α = 0.05) were done to determine whether the seed lots differed with respect to emergence and incidence of disease-like symptoms.

The experiments was organised according to Completely Random Design (CRD) using the six seed lots and eight repetitions of each. One-way analysis of variance (ANOVA) and comparison of means based on the Tukey test (α = 0.05) were done to find out whether the seed lots tested differed statistically significantly for each of the evaluated variables. The most important result from ANOVA are summarized in Table 1.

**Table 1:** Emergence of beans (cv. INTA Rojo) and the portion of emerged seedlings showing disease-like symptoms 15 days after planting under controlled conditions. Six bean storehouses belonging to different small farmers’ cooperatives were sampled in four regions in Nicaragua. Eight subsamples (50 seeds each) were taken from each store and planted under controlled conditions. Least significant difference of means for emergence = 10.4 (p = 0.00006; Tukey, α = 0.05).

Seed lot no.	Region	Mean emergence (%)	Emerged seedlings with symptoms (%)^a^
**1**	Boaco	24.9	35
**2**	Carazo	38.3	9
**3**	Carazo	24.3	48
**4**	Estelí	33.1	25
**5**	Estelí	30.0	39
**6**	Matagalpa	33.6	31

^a^Percentage of the emerged seedlings that showed disease symptoms, including cankers, stem or root lesions, necrosis, and/or wilting.

### Isolation of fungi

Eight samples (8 beans each) were taken from each seedlot and surface-sterilized by submerging first into 3% sodium hypochlorite solution for 10 min and then 70% ethanol for 3 min, followed by rinsing with sterile distilled water for 5 min and letting dry for a short while on sterile filter paper in a laminar flow cabinet. Two growth media were used for fungal isolation: potato dextrose agar (PDA) and nutrient agar (Merck Millipore) complemented with streptomycin (Sigma) at 50 mg/l [13]. Surface-sterilized beans were placed on growth medium in Petri dishes (Ø 10 cm), 8 beans per dish. Lids of Petri dishes were closed and sealed with Parafilm (Bemis), and the dishes were incubated at room temperature (25–30°C) in the dark for 4–7 days. As soon as fungal growth was observed on beans, mycelium was transferred with sterile forceps to fresh culture medium. As the fungus grew, single tips of mycelia were picked from the edge of the colony and transferred to fresh medium. The pure cultures of fungi thus obtained were stored at room temperature in the dark.

### Pathogenicity tests

**Fig 1:**
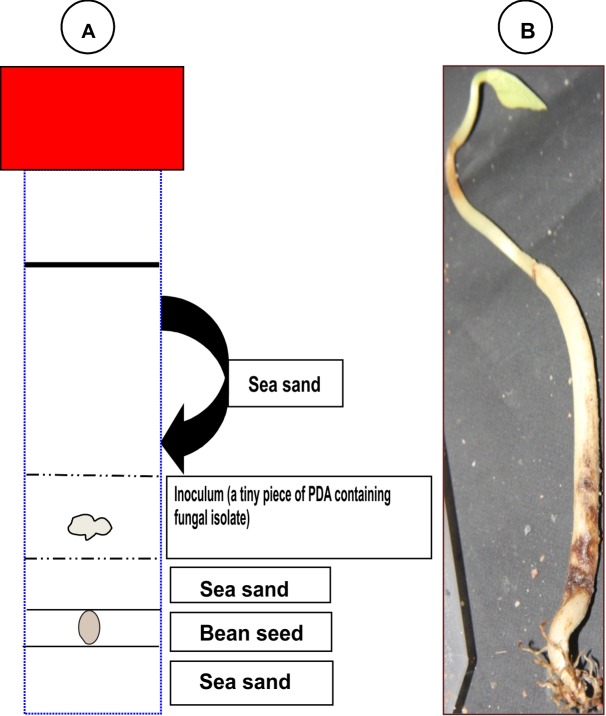
Layout of the pathogenicity test. (a) A pathogen-free pre-germinated lima bean was placed on a layer of sterilized, moist sea sand in a 50-ml sterile test tube. A layer (1 cm) of sand was added, and a piece of PDA containing hyphae of the fungus was placed on the sand. The tube was filled with sand up to 2 cm from the top, closed loosely with a cap, and incubated at 20°C under dim light in a growth room. (b) Seedlings reached a height of 10–12 cm (unless heavily damaged) and were observed for symptoms at 20 dpi. Symptoms shown in (b) were caused by *Macrophomina phaseolina*.

Pathogenicity of 113 fungal isolates on beans was assessed twice in two independent experiments, as described elsewhere [13]. There were four replicates (four tubes) and one non-inoculated control for each fungal isolate per experiment. Lima bean (*P*. *lunatus* L.) obtained from the former MTT Agrifood Research Finland (currently Natural Resources Institute) was used for pathogenicity tests because healthy seeds of ‘INTA Rojo’ or other common bean varieties grown in Nicaragua were not available. Wild forms of lima bean are of Mesoamerican and Andean origin and grow in Nicaragua. They are likely exposed to the same pathogens as cultivated common beans. Lima beans were surface-sterilized (as described above) and germinated on moist sterile filter paper in Petri dishes. Sterilized (autoclaved) sand (~10 ml) was transferred to a sterile plastic test tube (50 ml) and moistened with sterile water. A healthy germinated bean was placed on the layer of sand (Fig 1). More sand (10 ml) was added to cover the bean, after which a piece of PDA containing hyphae of the test fungal isolate was taken with a cork borer (Ø 5 mm) and placed on the sand. Finally, the test tube was filled with sterile sand and closed gently with a cap. Later, the cap was opened to allow emergence of the sprout.

Tubes were incubated at 20°C under dim light in a growth room. Pathogenicity of the fungal isolates was evaluated 20 days post-inoculation (dpi). Sand and the seedling were gently removed from the tube and symptoms recorded. To fulfill Koch’s postulates, pieces of symptomatic tissue were excised from the seedlings with a sterile scalpel, transferred to PDA, and fungal growth was monitored and identified with help of a microscope.

### DNA isolation and PCR amplification of the ITS regions

Mycelia were ground in liquid nitrogen and DNA isolated using the cetyltrimethylammonium bromide (CTAB) method [14] with minor modifications (CTAB extraction buffer: 2% w/v CTAB, 20 mM sodium EDTA, 100 mM Tris-HCl, pH 8.0, and 1.4 M NaCl). The internal transcribed spacer 1 (ITS1) and 2 (ITS2) regions of the rRNA genes were amplified using universal primers (ITS-1: 5’-TCCGTAGGTGAACCTCCGG-3’; ITS-4: 5’-TCCTCCGCTTATTGATATGC-3’) specific for the flanking 18S and 28S rRNA genes in fungi [15]. Each PCR reaction (50 μl) contained 10 μl of 5× Phusion High Fidelity reaction buffer (Finnzymes), 1 μl of dNTPs (10 mM), 1.5 μl of 20 μM primers (ITS-1 and ITS-4), 0.25 μl of Phusion High Fidelity DNA polymerase (2 U/μl, Finnzymes) and 250 ng of DNA template in nuclease-free water. Amplification was carried out in a thermal cycler (Eppendorf Mastercycler Gradient) using the following program: initial denaturation at 98°C for 1 min, followed by 34 cycles of denaturation at 98°C for 15 s, annealing at 63°C for 15 s, extension at 72°C for 15 s, and final extension at 72°C for 5 min and hold at 10°C. Reaction products were analyzed by electrophoresis on 1% agarose gels. The expected size of the PCR product amplified by the ITS-1/ITS-4 primer pair was ~600 nt [15].

### DNA sequencing

PCR products were purified using the EZNA gel extraction kit (Omega Bio-Tek), exonuclease I of *Escherichia coli* (EXOI) (Fermentas), and either calf intestine alkaline phosphatase (CIAP) (Fermentas) or shrimp alkaline phosphatase (SAP) (Fermentas). To 40 μl of PCR product, 4 μl of EXOI and 8 μl of CIAP (or SAP) were added, mixed well, and incubated at 37°C for 15–20 min and at 75–80°C for 20 min. Direct sequencing of purified PCR products (15 μl) was done using the primer ITS-1 at Haartman Institute, University of Helsinki, Finland. The sequenced region included partial ITS1, the 5.8S rRNA gene, entire ITS2, and part of the 28S rRNA gene.

### Species identification and sequence comparisons

Taxonomic keys [16–19], Index Fungorum (www.indexfungorum.org), and species descriptions linked to the Taxonomy Browser of NCBI (www.ncbi.nlm.nih.gov/Taxonomy/Browser/) were consulted to identify the fungi. Morphological characters of fungi were assessed under a light microscope (Leica), and when necessary, hyphae and spores were stained with lactophenol cotton blue (20 g deionized water, 20 g phenol, 20 g lactic acid, 40 g glycerol, 0.05 g cotton blue).

Representative sequences determined in this study were deposited to the NCBI sequence database (S1 Table). BLAST (http://blast.ncbi.nlm.nih.gov/Blast) was used to compare the nucleotide sequences of the PCR products including partial ITS1, 5.8S, and ITS2 (~450 nt), with fungal sequences available in the NCBI database. Sequences were aligned using CLUSTAL-X. Nucleotide identities between sequences were computed using the CLUSTAL-W procedure. Phylogenetic analyses were carried out with the neighbor-joining method using 1000 replicates and the Kimura two-parameter model as implemented in MEGA version 5 [20].

## Results

### Emergence and growth of bean seedlings

Emergence of bean seedlings was low (24–38%) regardless of the source storehouse as observed 15 days after planting (Table 1). However, differences in emergence were significant between some storehouses (one-way analysis of variance, p = 0.00006). The poorest emergence was observed with beans from Boaco and one storehouse in Carazo, whereas beans from the other storehouse in Carazo showed the best emergence (Table 1).

**Fig 2:**
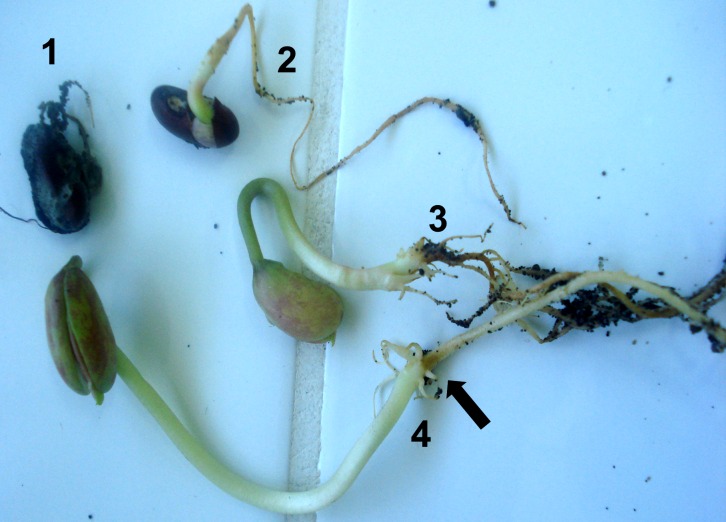
Types of symptoms in beans sampled from storehouses and grown in sterilized soil under controlled conditions. Plants were inspected 15 days after planting. 1, Early death of roots and the seedling; 2, root rot and poor emergence of the seedling; 3, necrosis in roots and poor growth of the seedling; 4, discoloration (mild necrosis) at the neck of the tap root (indicated by the black arrow).

All emerged seedlings were inspected for symptoms. Depending on the source, 9–48% of the emerged seedlings displayed disease-like symptoms, but differences between seed lots were not significant (p = 0.130). Lethal-to-mild necrosis or different levels of discoloration were observed on roots, and cankers and necrotic areas were observed in shoots. The most severe symptoms were associated with poor growth of seedlings (Fig 2). Beans that failed to emerge were also inspected, and most were found to be rotten—often covered by fungal mycelia. Some soft and rotten beans had an unpleasant odor, suggesting bacterial infection, which was not studied further.

### Pathogenicity of fungal isolates in different phenogroups

In total, 133 fungal isolates were obtained from surface-sterilized beans of the six seed lots and tested for pathogenicity on lima beans. The results of two independent experiments were consistent in showing that 87 fungal isolates caused symptoms on bean seedlings. Typical symptoms included cankers, necrosis, growth decline, dieback, or rot at 20 dpi. No obvious symptoms were detected in seedlings growing from beans inoculated with the remaining 45 isolates or those that were mock-inoculated using a piece of PDA without fungus.

All the phenotypically similar fungal isolates were designated to a phenogroup based on the observed morphological and growth characteristics. Eight distinguishable phenogroups were identified (Table 2). Only the pathogenic isolates were considered for further study.

**Table 2:** Phenotypic characteristics and pathogenicity of the fungal isolates from common bean (cv. INTA Rojo). Phenotypic features of fungal isolates grown on nutrient agar were compared, and the phenotypically similar isolates were designated to the same ‘phenogroup’. Pathogenicity was tested on lima beans under controlled conditions.

Pheno-group	Phenotypic characters	No. of pathogenic isolates out of total no. of isolates	Symptoms caused by pathogenic isolates	Identification of pathogenic isolates^a^
I	Colonies fast growing; mycelia whitish to yellow, pink or orange. Conidia and chlamydospores observed.	32/53	Necrosis and cankers on stems, wilting, seed and stem rot, decline, poor growth.	*Fusarium chlamydosporum* (Wollenv.), *F*. *equiseti* (Corda) Sacc., *F*. *incarnatum* (R) Sacc.
II	Colonies grey to black, homogeneous, fast growing. Proliferation and aggregation of hyphae, microsclerotia. Some isolates produce aerial mycelium.	10/14	Charcoal rot, necrotic lesions on stems, root rot, growth decline, decay of stems, black sclerotia.	*Macrophomina phaseolina* (Tassi) Goidanich
III	Colonies greyish to black, aerial mycelium, shiny grey; dense and feathery growth. Mature two-celled dark-brown conidia with striations. Conidia hyaline, oval shape.	16/22	Dieback, decay, cankers on stems; plant decline.	*Lasiodiplodia theobromae* (Pat.) Griffon & Maublanc
IV	Colonies grayish-brown to brown- eddish; well-formed acropetal conidia in chains.	2/2	Dark or brown-dark lesions on stems, softer and thinner stem, root rot and blight.	*Corynespora cassiicola* (Berk. & M.A. Curtis) C.T. Wei
V	*Colletotrichum* genus subdivided:	8/8		
	Colonies grey-olive, white or grey-dark brown and circular in shape with perithecia and acervuli; conidia cylindrical and obtuse.	6	Necrosis, brown lesions, spots, cankers on stems, seed rot, soft stem and leaf blight, dieback.	*Collectotrichum gloeosporioides* (Penz.) Penz. & Sacc.
	Colonies brown, dense mycelial growth with copious acervuli.	2	Small dark spot on the stem, discoloration of roots and dark spots on the cotyledon.	*C*. *capsici* Syd E.J. Butler & Bisby
VI	Colonies fast growing, flat, dense, downy; white at the periphery and green at center; blue green conidia.	13/26	Wilting, lesions on stems, soft stem, rot.	*Penicillium citrinum* Link
VII	Young colonies yellowish-green or white, later dark green; downy. Conidiophore and vesicle globose with green conidia.	4/4	Dark roots, dark-brown lesions on stems, soft stem, necrosis.	*Aspergillus flavus* Link
VIII	Colonies of floc form, dense white mycelium. Black pycnidia.	2/4	Stem canker, root rot, decline and leaf spots.	*Diaporthe* sp. Nitschke, anamorph *Phomopsis* sp. Sutton

^**a**^Tentative identification at genus or species level was done according to taxonomic keys [16–19] and augmented by analysis of the ITS sequences.

**Fig 3:**
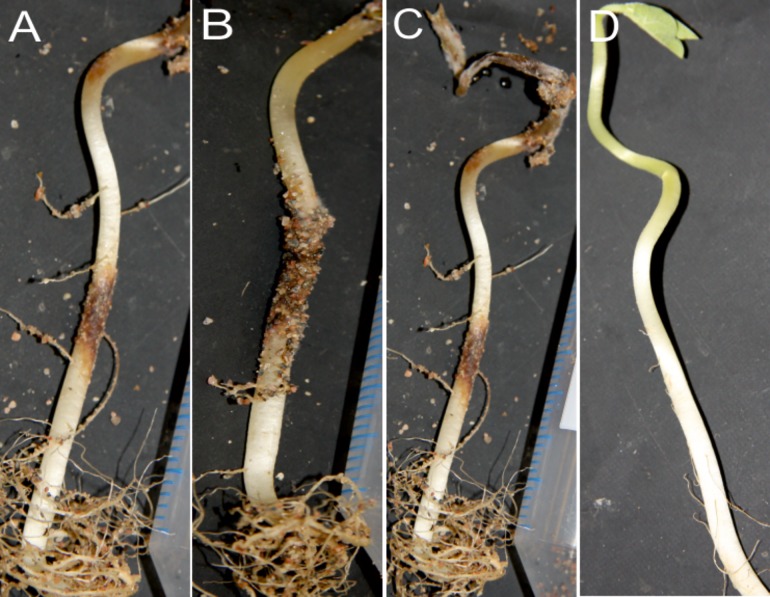
Symptoms of lima bean seedlings caused by fungal isolates of phenogroup IV. (a) to (c), inoculated plants at 20 dpi, and (d), non-inoculated control. Necrosis in the middle of the stem corresponds to the position of inoculum placed in the test tube. All inoculated seedlings display severe apical necrosis.

We found that the pathogenic isolates within phenogroups were uniform in terms of the types of symptoms they caused in bean seedlings. Isolates of phenogroup I caused root rot, lesions on the stem, and poor growth of seedlings (S1A Fig), whereas no damage was observed in the non-inoculated controls. Isolates of phenogroup II induced charcoal rot, dark lesions on stems, and root rot (S1B Fig). Isolates of phenogroup III (S1C Fig) and IV (Fig 3) induced severe symptoms including dieback, decay, and cankers on stems and roots of the inoculated bean seedlings.

**Fig 4:**
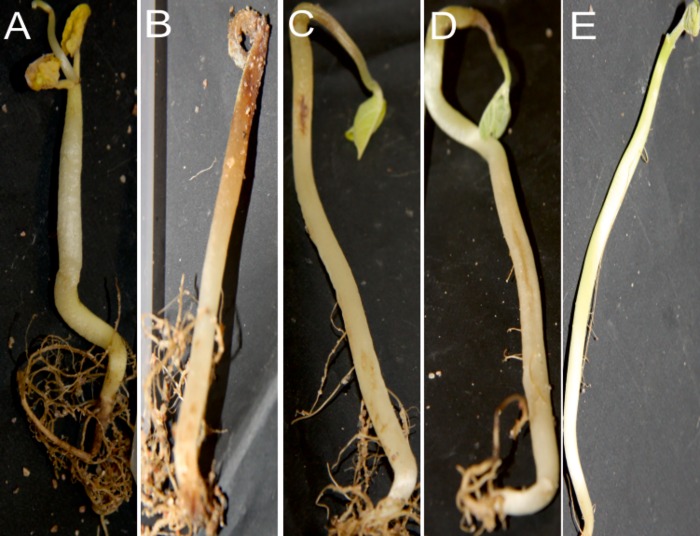
Symptoms of lima bean seedlings caused by fungal isolates of phenogroup VIII. (a) to (d), inoculated plants at 20 dpi, and (e), non-inoculated control. (a) and (b), necrotic roots and whitish mycelia growing on the stem; necrosis at apex of the seedling. (c) and (d), cankers on stem and wilted apex of the seedling.

Isolates in phenogroup V induced symptoms of anthracnose, including small dark spots on the stem, discoloration of roots, and dark spots or small dark-brown-to-black lesions on cotyledons (S1D Fig). The isolates of the phenogroups VI (S1E Fig) and VII (S1F Fig) caused merely mild symptoms such as discoloration of the stem, whereas isolates of phenogroup VIII caused cankers and decay (Fig 4). Fungi were re-isolated from the inoculated, symptomatic plant tissue to PDA, grown, and identified, thus fulfilling Koch’s postulates.

### Species of pathogenic fungi detected in seed beans and confirmed in lima bean

Isolates placed to phenogroup I were morphologically similar to *Fusarium* spp. (Phylum Ascomycota; Class Sordariomycetes; Order Hypocreales; Family Nectriaceae) (S2A Fig). ITS sequences were 99–100% identical to those of *F*. *chlamydosporum* (Wollenv.), *F*. *equiseti* (Corda) Sacc. *sensu* Gordon, or *F*. *incarnatum* (R.) Sacc. *Fusarium* spp. were detected in all seed lots except lot no. 2 from Carazo.

In phenogroup II, the ITS sequences were 99–100% identical to *Macrophomina phaseolina* (Tassi) Goid (anamorph or synonymous with *Rhizoctonia bataticola* Taub.) (Phylum Ascomycota; Class Ascomycetes; Order Incertae sedis; Family Incertae sedis). *M*. *phaseolina* was detected in Boaco, Carazo, and Matagalpa.

**Fig 5:**
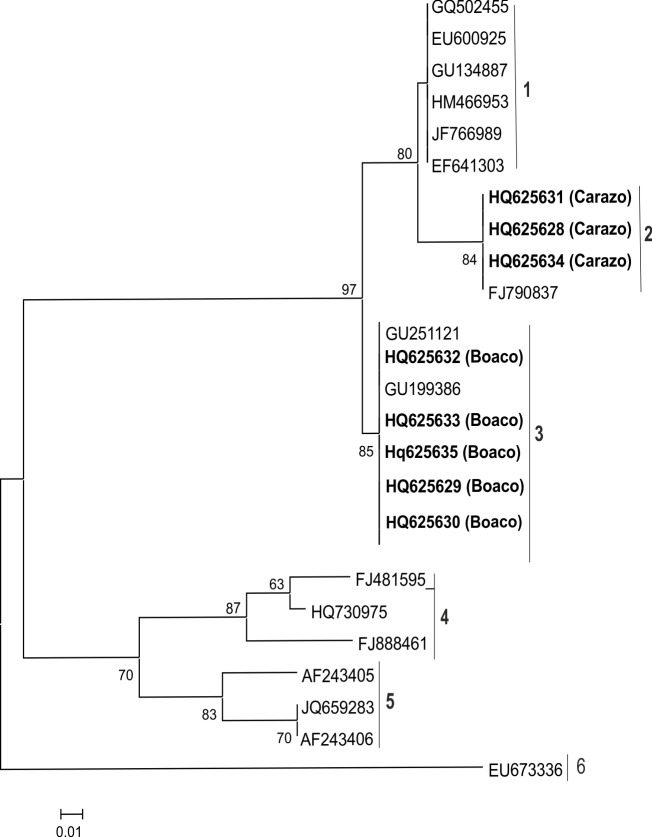
Phylogenetic grouping of the fungal isolates in phenogroup III. Partial ITS1 and the whole 5.8S and ITS2 sequences (~450 nt) of the fungi isolated from beans grown in Boaco and Carazo (bold letters) were included in the phylogenetic analysis with sequences of *Lasiodiplodia theobromae* obtained from sequence databases. Numbers at branches represent bootstrap values of 1000 replicates. Only bootstrap values ≥70% are shown. Scale indicates Kimura units in nucleotide substitutions per site.

Isolates of phenogroup III were detected in the seed lot from Boaco and one seed lot (no. 3) from Carazo. Morphological features were similar to *Lasiodiplodia* spp. (Phylum Ascomycota; Class Dothideomycetes; Order Botryosphaeriales; Family Botryophaeriaceae), which was consistent with high ITS sequence identities (99%) compared with *Lasiodiplodia theobromae* (Pat.) Griffon & Maublanc, and the teleomorph *Botryosphaeria rhodina* (Berk, & Curt. v. Arx) Penz. (Fig 5, S2B Fig). In the phylogenetic analysis, sequences of *L*. *theobromae* isolates obtained from the databank fell in five distinguishable phylogenetic clusters supported by bootstrap values ≥70%. Sequences of the isolates differed between Boaco and Carazo but were identical within each region (Fig 5).

Sequences of phenogroup IV isolates were most closely related to *Corynespora* spp. (Phylum Ascomycota; Class Dothideomycetes; Order Pleosporales; Family Corynesporascaceae), and all were identical to *C*. *cassiicola* (Berk. & M.A. Curtis) C.T. Wei. Two pathogenic isolates were detected in Carazo (seed lot no. 2).

**Fig 6:**
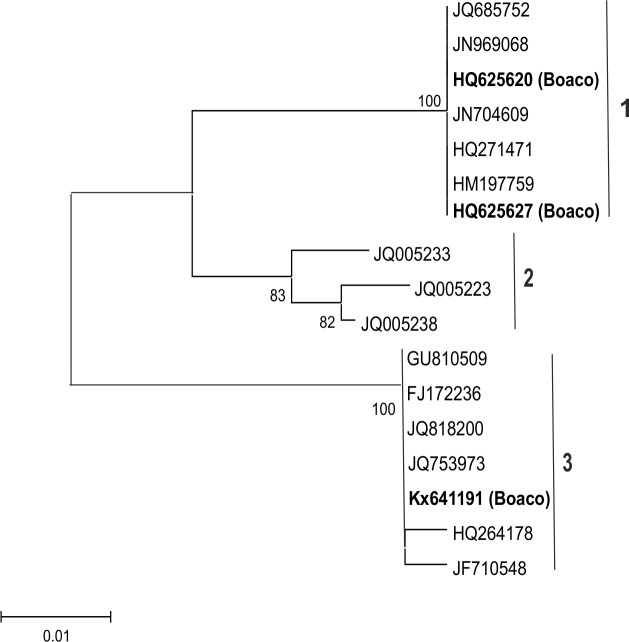
Phylogenetic grouping of the fungal isolates in phenogroup V. Partial ITS1 and the whole 5.8S and ITS2 sequences (~450 nt) of the fungi isolated from beans grown in Boaco (bold letters) were included in the phylogenetic analysis with sequences of *Colletotrichum capsici* (clade 1), *C*. *gloeosporioides* (clade 3), *C*. *parsonsiae* (JQ005233), *C*. *petchii* (JQ005223), and *C*. *constrictum* (JQ00538) (clade 2) obtained from sequence databases (S1 Table). Numbers at branches represent bootstrap values of 1000 replicates. Only bootstrap values of ≥70% are shown. Scale indicates Kimura units in nucleotide substitutions per site.

Phenogroup V contained isolates related to *Colletotrichum* species. The sequence of one isolate from Boaco was identical to isolates of *C*. *gloeosporioides* (Penz.) Penz. & Sacc. [teleomorph *Glomerella cingulata* (Stoneman) Spaulding & von Schrenk] (Phylum Ascomycota; Class Sordariomycetes; Order Incartae sedis; Family Glomellaceae; Genus *Glomerella*). Sequences of the four other isolates were identical to *C*. *capsici* (Syd) E.J. Butler & Bisby (Phylum Ascomycota; Class Sordariomycetes; Order Phyllacharales; Family Phyllachoraceae). Because the sequences of all four isolates were identical, only two of them were included in the phylogenetic analysis (Fig 6). In total, seven *C*. *capsici* isolates were detected in samples from Boaco and Matagalpa.

Phenogroup VI included *Penicillium* spp. detected in all six regions. The ITS sequences were 99% identical to *Penicillium citrinum* (Link). Phenogroup VII contained isolates with ITS sequences identical to *Aspergillus flavus* (Link). They were detected in Estelí (seed lot no. 5) and Matagalpa. *Aspergillus* spp. and *Penicillium* spp. belong to Phylum Ascomycota; Class Eurotiomycetes; Order Eurotiales; Family Trichocomaceae.

Phenogroup VIII isolates showed 99–100% identity to fungi in the genus *Diaporthe* Nitschke (anamorph *Phomopsis*, Sutton) belonging to Phylum Ascomycota; Class Sordariomycetes; Order Diapothales; Family Diaporthaceae. One pathogenic isolate was detected in the seed lot from Boaco and seed lot no. 5 from Estelí.

## Discussion

Seedborne pathogenic fungi in beans used for seeds reduce germination, emergence, growth, and yield, whereas in beans used for food they can reduce the nutritional value or produce toxins making the beans unsuitable for consumption [21]. The fungi can be transmitted as contaminants that adhere to the seed coat, or infect the seed, which is considered as the main mechanism of seed-mediated transmission. This work showed that germination in seedlots of common bean (‘INTA Rojo’) from four important bean production areas in Nicaragua was always less than 40% and as low as 16%, which is potentially disastrous for the farmers. Subsequently, we detected 87 pathogenic fungal isolates from surface-sterilized beans in six seed lots of INTA Rojo. Results showed that those seed lots that exhibited better emergence gave rise to a larger proportion of healthy and vigorous seedlings, whereas poor emergence was associated with a larger proportion of seedlings that emerged but were abnormal, grew poorly, and/or were affected by disease-like symptoms.

The pathogenic fungi isolated in this study were classified phenotypically to eight distinguishable groups (phenogroups) based on growth and morphological characteristics and further identified by analysis of the ITS1 and ITS2 sequences [22,23]. The most common fungi among the pathogenic isolates were *Fusarium* (*F*. *chlamydosporum*, *F*. *equiseti*, *F*. *incarnatum*), *L*. *theobromae*, *P*. *citrinum*, and *M*. *phaseolina*. These fungi are discussed individually below, and management options are presented together at the end.

*Fusarium* spp. were detected in seedlots in all four regions surveyed in Nicaragua. *Fusarium* species are soil-borne fungi that can cause rot of the root, stem, and fruit or vascular wilt in a wide range of crop plants, and they survive as saprophytes [24]. The wide range of different races contributes to the taxonomic complexity [25,26]. Mycotoxin production by *Fusarium* spp. is of concern to human and animal health in many field crops, including common beans [27–29]. There is scant previous information about seedborne infections of *F*. *incarnatum* in common beans or its pathogenicity on common bean seedlings. However, *F*. *equiseti* is known to infect many forms of bean, including bush bean (*P*. *lunatus*), kidney bean and haricot bean (*P*. *vulgaris*) [30,31], as well as faba bean (*Vicia faba* L.), pea (*Pisum sativum* L.), lentil (*Lens culinaris* L.) [31], cowpea (*Vigna unguiculata* L.) [32], soybean [*Glycine max* (L.) Merr.] [33], and mung bean [*Vigna radiata* (L.) R. Wilczek.] [30]. *F*. *equiseti* occurs mainly in tropical and subtropical regions, but it has also been found in temperate areas in Europe and North America [34,35]. It is highly adaptable to many cropping systems and is capable of infecting seeds, roots, tubers, and fruit [36]. *F*. *incarnatum* can infect other crops, such as *Capsicum annum* L. [37] and *Ziziphus jujube* Mill. [38]. The third species, *F*. *chlamydosporum*, has been isolated previously from soil, beans, and maize roots in Kenya [39]. Problems with *Fusarium* spp. are experienced in common bean production also elsewhere in the Central American region. In Cuba, half of the seedlots surveyed for fungi were found to contain *Fusarium* spp. [40], including *F*. *solani* f. sp. *phaseoli* causing substantial yield losses in common bean crops in many regions of Mexico [41]. Studies on disease epidemiology and genetic diversity of *Fusarium* spp. that infect common bean have been initiated in Mexico [41] and are needed in Nicaragua.

*L*. *theobromae* was rather abundant in beans harvested in Boaco and Carazo. Genetic diversification of this species was apparent with two clusters being identified in the phylogenetic analysis (Fig 5). Genetic differences correlated geographically, because the isolates from Boaco and Carazo were assigned to different clusters. Common bean seedlings and seedlings of lima bean displayed similar symptoms of dieback, decay and cankers following infection with *L*. *theobromae*. No difference in pathogenicity was observed between the two genetically distinguishable groups of isolates. Little is known about diseases of common bean caused by *L*. *theobromae*, although it causes disease in more than 500 plant species [42–44] and is endemic to tropical and subtropical regions. It can also colonize plant tissues without any visible symptoms of infection and live as an endophyte or saprophyte [43,45–48]. The change from a non-pathogenic lifestyle to a disease-causing pathogen may be associated with host stress [45,48,49]. The spores of *L*. *theobromae* are disseminated by rain and wind [50]. Use of fungicides and resistant/tolerant cultivars may be helpful to decrease the occurrence of *L*. *theobromae* infections [51].

*M*. *phaseolina* was detected in beans harvested from three of the four surveyed regions. In this species as well, genetic diversification was observed, resulting in placement of the isolates into three clades based on the phylogenetic analysis (data not shown). Besides several Nicaraguan isolates, one clade included isolates of *M*. *phaseolina* from *Vigna radiata* L. (China), *Fragaria* × *ananassa* (Spain), *Pisum sativum* (Australia), and *Fraxinus* sp. (USA). These results are consistent with previously reported variation in morphology and virulence among isolates of *M*. *phaseolina* in plants comprising common bean, soybean, and other crops [51,52]. According to Su et al. [53] the host specialization of *M*. *phaseolina* is apparent in corn but not in sorghum, cotton, or soybean. Indeed, *M*. *phaseolina* is one of the commonest pathogens of common bean and considered a polyphagous pathogen able to infect several hundred plant species [54–56]. This fungus survives in the soil as microsclerotia and in the debris of infected plants. Large populations of *M*. *phaseolina* in the soil may develop when the host is susceptible and cropped in consecutive years, and the pathogen is redistributed by tillage practices. Furthermore, some strains of *M*. *phaseolina* have adapted to certain types of climate and soil [57]. Increased salinity of soil stimulates infection and may increase disease severity [56].

Seven isolates of *Colletotrichum capsici* (Boaco and Matagalpa) and one isolate of *C*. *gloeosporioides* (Boaco) were detected in the seedlots. ITS sequences of *C*. *capsici* isolates from Boaco and Matagalpa were identical to each other and to those from pepper in Malaysia, India, and Mexico (Fig 6; S1 Table). The ITS region of the *C*. *gloeosporioides* isolate from Boaco was identical to an isolate characterized from common bean in Brazil, and also identical to an isolate from soybean (Taiwan) and lemon (New Zealand). Hence, genetically similar isolates seem to be widely distributed and able to infect a wide range of host species. *C*. *capsici* is typically a pathogen of pepper (*Capsicum* spp.), but all *C*. *capsici* isolates (and the *C*. *gloeosporioides* isolate) detected in seed beans in our study were found to cause cankers and severe wilting in inoculated lima bean seedlings. Bean anthracnose is typically caused by *Colletotrichum lindemuthianum* (Sacc. & Magnus) Briosi & Cavara and considered one of the most severe diseases in beans. In navy bean, for example, infection of 7% of bean seeds was sufficient to cause statistically significant yield losses [58]. Anthracnose damages foliage, stems, and pods and reduces germination as well as product quality and yield. In the absence of susceptible host plants, *Colletotrichum* spp. survive over growth seasons as mycelia on infested crop residues as saprophytes, or in infected seeds. Plants can be infected at any growth stage. Symptoms are more obvious in mature plants and under disease-conducive moist conditions [9,59,60].

*Corynespora cassiicola* was detected in one seedlot sampled in Carazo and its ITS sequence was identical to many reference sequences of this species retrieved from the NCBI database. While common bean is indeed a host for *C*. *cassiicola* and suffers from target spot disease caused by the fungus [61], our study seems to be one of the few showing that *C*. *cassiicola* is not eliminated by surface sterilization of seed beans and is hence a truly seedborne pathogen in this species. Furthermore, the two characterized isolates caused very severe symptoms in the inoculated lima bean seedlings. Host species adaptation is suggested by studies showing that the most virulent isolates of *C*. *cassiicola* on common bean are those that have been isolated from that species, as compared with isolates from other crops such as basil, cowpea, cucumber, papaya, soybean, sweetpotato, or tomato [61]. *C*. *cassiicola* is an aggressive facultative parasite able to infect many legume species and considered one of the most damaging pathogens of soybean crops in Brazil [62] and Korea [63]. *C*. *cassiicola* sporulates on plant debris and also survives in soil without plant residues [64,65]. The conidia infect leaves and stem. The fungus requires rather high soil temperature (15–20°C) and moisture for infection and disease development. The disease cycle is completed in 7–10 days [66].

Two isolates of *Diaporthe* sp. (synonym *Phomopsis* sp.) [67], one each from Boaco and Estelí, were characterized from the common bean seedlots. When they were used to inoculate lima bean seedlings, white mycelia developed as described in soybeans that suffer from stem and pod blight disease following infection with *Diaporthe phaseolorum* var. *sojae*/*Phomopsis sojae* [29]. Furthermore, severe symptoms of necrosis and wilting developed in the inoculated lima bean seedlings. *Diaporthe* spp. are pathogens of many different plant species and cause seed rot, stem cankers, lesions, and pod blight [68], but in common beans they are simply endophytes [69]. Therefore, it is remarkable that the two isolates of *Diaporthe* from seedlot of ‘INTA Rojo’ caused severe disease symptoms in common bean seedlings (Fig 4). Recently, *D*. *masirevicii* and *D*. *miriciae* were found associated with cankers on soybean and mung bean plants in Australia [68]. Analysis of the ITS regions alone is insufficient to identify the species in the genus *Diaporthe* [69]. It therefore seems warranted to further characterize the pathogenic isolates described in this study using, e.g., multilocus phylogenetic analysis [69].

Two genera, *Penicillium* and *Aspergillus*, were found to be associated with post-harvest losses in ‘INTA Rojo’. *Penicillium* grew out from a few seed beans of all seedlots, in spite of prior surface-sterilization, and isolates caused mild necrotic symptoms on inoculated bean seedlings. ITS sequences identified the species as *P*. *citrinum*. A survey of seedlots in Taiwan and Ontario also revealed a number of different *Penicillium* spp. in surface-sterilized beans, albeit not *P*. *citrinum* [27]. Four isolates of *Aspergillus flavus* were obtained from two seedlots (Estelí and Matagalpa). All of them caused cankers and mild necrosis on inoculated bean seedlings, consistent with a previous study that reported necrosis on roots and stem as well as leaf spots in various legumes caused by this species [70]. Post-harvest rotting of cereal grains and legumes causes large economical losses as it may destroy 10–30% of the yield—or even higher portions in developing countries [29]. However, the most worrying aspect about *A*. *flavus* is its potential in many different crops to produce aflatoxins known to be among the most potent carcinogens of biological origin [71].

Better control of the pathogenic fungi detected in common bean is needed in Nicaragua for improvement of bean production, reduction of yield losses and minimizing health risks caused by fungal toxins and allergens. All fungi found to infect common bean in this study can infect also a wide range of other plant species that act as possible pathogen reservoirs for infection of common bean crops. The efficient dissemination of conidia by rain and wind constitutes another challenge for control of the pathogens. Resistant cultivars and integrated pest management play important roles in preventing seedborne fungal diseases of common bean [72]. For example, control of charcoal rot caused by *M*. *phaseolina* in beans depends on crop management comprising the use of resistant cultivars, crop rotation, avoiding too dense a canopy, controlling soil moisture and water stress with irrigation and tillage practices, and, potentially, the use of biological control [73,74]. *Trichoderma* spp. have shown good antagonistic capacity against anthracnose [75] caused by *C*. *capsici* that was one of the pathogens detected in common bean in this study. Crop rotation requires careful planning. For example in soybean production, crop rotation with maize is essential for reducing infection pressure by *C*. *cassiicola* [76], but it is not suitable for control of *Diaporthe* spp., because maize supports this genus from one cropping season to the next [68]. Control of *Fusarium* spp., *L*. *theobromae*, *C*. *capsici* [72,75] and other fungi with fungicides to prevent mycotoxin production and excessive yield losses is possible but causes another health risk to farmers and consumers [28,29]. Finally, the rather common occurrence of *Penicillium* and *Aspergillus* in the stored beans in Nicaragua causes a risk for exposure to mycotoxins and allergens and calls for better management of bean crops in the field and improved post-harvest practices [22,77].

In conclusion, this survey of pathogenic fungi in seedlots of common bean, which focused on the nationally important cultivar ‘INTA Rojo’ grown in the four main bean production regions in Nicaragua, revealed eight fungal genera harmful to seed quality as judged on their ability to infect and damage naturally infected common bean seedlings and inoculated lima bean seedlings. Many of these fungi are well known pathogens that cause seed decay, root rot, stem cankers, wilting, necrosis, and/or death of infected bean plants; for example, *Fusarium* spp., *Penicillium* spp., and *A*. *flavus* are the predominant species detected in common bean in Cuba [40]. On the other hand, many of the seedborne pathogenic fungi detected in ‘INTA Rojo’ were previously unreported in Nicaragua, and reports on occurrence of some, such as *F*. *incarnatum*, *L*. *theobromae*, *C*. *cassiicola*, and *Diaporthe*, as seedborne pathogens of common bean are rare elsewhere. The incidences of the pathogenic fungi differed between seedlots, which calls for further study to understand the basis of differences in seed quality and use of the results to improve handling and storage conditions of seed beans. The results provide a knowledgebase for further development of diagnostic tools for seed health inspection and seed certification. It is also important to continue studies on epidemiology, ecology, and control of the pathogenic fungi of common bean in the field and to improve control of the diseases by integrated crop management and use of certified seeds and resistant varieties.

## Supporting Information

S1 Table Fungal ITS sequences determined in this study, and the reference sequences retrieved from databases and used for comparison. (DOC)

S1 Fig Symptoms caused by the fungi isolated from seedlots of common bean in Nicaragua and inoculated to healthy lima beans under controlled conditions. Photographs were taken at 20 dpi. Panel (a) *Fusarium* spp., (b) *Macrophomina phaseolina*, (c) *Lasiodiplodia theobromae*, (d) *Penicillium citrinum*, (e) *Colletotrichum capsici* (photos A and B) and *Colletotrichum gloeosporioides* (photos C and D), and (f) *Aspergillus flavus*. The right-most photograph in each panel shows the mock-inoculated control (photograph E in panels a, c and e, and photograph D in panels b, d and f).
(TIF)

S2 Fig Mycelia and spores of pathogenic fungi isolated from seed lots of common bean in Nicaragua and photographed following growth on PDA agar for 12 and 13 days. For microscopy, hyphae and spores were stained with lactophenol cotton blue. (a) *Fusarium equiseti* (NCBI accession no. HQ625615): A, mycelia; B, characteristic sickle-like spores. (b) *Lasiodiplodia theobromae* (HQ625630): A, mycelia; B, hyphae including mature two-celled dark brown conidia with striations (arrowhead). (c) *Colletotrichum gloeosporioides* (KX641191): A, mycelia; B, spores.
(TIF)
