# Supplementary material for: Seedborne Pathogenic Fungi in Common Bean (Phaseolus vulgaris cv. INTA Rojo) in Nicaragua
Source: PLoS One. 2016 Dec 20;11(12):e0168662. doi: 10.1371/journal.pone.0168662 (PMC5173241; doi:10.1371/journal.pone.0168662)
Supplement: S1 Table — (DOC) [file pone.0168662.s001.doc]

**S1 Table**. Sequences of the ITS regions determined in this study and the reference sequences retrieved from databases and used for comparison.

| **Fungal species** | **Geographical origin** | **Sequence accession no.** | **Host** | **Reference** |
| --- | --- | --- | --- | --- |
| *Aspergillus flavus* | Estelí (NI) | KX641192 | *Phaseolus vulgaris* | This study |
| *Colletotrichum capsici* | Boaco (NI) | HQ625620 | *P. vulgaris* | This study |
| *C. capsici* | Boaco (NI) | HQ625622 | *P. vulgaris* | This study |
| *C. capsici* | Boaco (NI) | HQ625624 | *P. vulgaris* | This study |
| *C. capsici* | Matagalpa (NI) | HQ625627 | *P. vulgaris* | This study |
| *C. gloesporiodes* | Boaco (NI) | KX641191 | *P. vulgaris* | This study |
| *Corynespora cassiicola* | Carazo (NI) | HQ625619 | *P. vulgaris* | This study |
| *Diaporthe sp.* | Estelí (NI) | KX641190 | *P. vulgaris* | This study |
| *Fusarium chlamydosporum* | Estelí (NI) | HQ625617 | *P. vulgaris* | This study |
| *F. chlamydosporum* | Carazo (NI) | HQ625613 | *P. vulgaris* | This study |
| *F. chlamydosporum* | Carazo (NI) | HQ625609 | *P. vulgaris* | This study |
| *F. chlamydosporum* | Estelí (NI) | HQ625618 | *P. vulgaris* | This study |
| *F. equiseti* | Boaco (NI) | HQ625606 | *P. vulgaris* | This study |
| *F. equiseti* | Boaco (NI) | HQ625611 | *P. vulgaris* | This study |
| *F. equiseti* | Boaco (NI) | HQ625615 | *P. vulgaris* | This study |
| *F. equiseti* | Boaco (NI) | HQ625642 | *P. vulgaris* | This study |
| *F. equiseti* | Matagalpa (NI) | HQ625608 | *P. vulgaris* | This study |
| *F. incarnatum* | Matagalpa (NI) | HQ625610 | *P. vulgaris* | This study |
| *F. incarnatum* | Boaco (NI) | HQ625612 | *P. vulgaris* | This study |
| *F. incarnatum* | Boaco (NI) | HQ625616 | *P. vulgaris* | This study |
| *F. incarnatum* | Boaco (NI) | HQ625607 | *P. vulgaris* | This study |
| *L. theobromae* | Carazo (NI) | HQ625631 | *P. vulgaris* | This study |
| *L. theobromae* | Carazo (NI) | HQ625634 | *P. vulgaris* | This study |
| *L. theobromae* | Carazo (NI) | HQ625636 | *P. vulgaris* | This study |
| *L. theobromae* | Boaco (NI) | HQ625629 | *P. vulgaris* | This study |
| *L. theobromae* | Boaco (NI) | HQ625630 | *P. vulgaris* | This study |
| *L. theobromae* | Boaco (NI) | HQ625632 | *P. vulgaris* | This study |
| *L. theobromae* | Boaco (NI) | HQ625633 | *P. vulgaris* | This study |
| *L. theobromae* | Boaco (NI) | HQ625635 | *P. vulgaris* | This study |
| *L. theobromae* | Carazo (NI) | HQ625628 | *P. vulgaris* | This study |
| *M. phaseolina* | Boaco (NI) | HQ625637 | *P. vulgaris* | This study |
| *M. phaseolina* | Boaco (NI) | HQ625638 | *P. vulgaris* | This study |
| *M. phaseolina* | Boaco (NI) | HQ625640 | *P. vulgaris* | This study |
| *M. phaseolina* | Carazo (NI) | HQ625641 | *P. vulgaris* | This study |
| *Penicillium citrinum* | Carazo (NI) | KX641193 | *P. vulgaris* | This study |
| Reference sequences obtained from NCBI database | | | | |
| *Aspergillus flavus* | India | KF031021 | *Syzygium cumini* | [57] |
| *A. flavus* | India | KC131548 | Arid soil | [26] |
| *A. flavus* | Malaysia | JX501383 | Soil | [52] |
| *A. flavus* | China | JN831610 | Unknown | [58] |
| *A. flavus* | Colombia | KF240822 | Wastelands | [20] |
| *Aspergillus flavus* | Egypt | KM504167 | *Solanum nigrum* | [16] |
| *Botryosphaeria tsugae* | unknown | AF243405 | Unknown | [62] |
| *Colletotrichum capsici* | India | JN704609 | *Capsicym annuum* cv. Byadagi | [49] |
| *C. capsici* | India | HQ271471 | *Capsicum annuum* | [48] |
| *C. capsici* | Mexico | JN969068 | *Carica papaya* | [54] |
| *C. capsic* | India | HM97759 | *Capsicum annuum* (Chilli) | [11] |
| *C. capsici* | Mexico | JN969068 | *Carica papaya* | [52] |
| *C. capsici* | India | HM197759 | *Capsicum annuum* (Chilli) | [11] |
| *C. capsici* | Malaysia | JQ685752 | *Capsicum annuum* | [30] |
| *C. cassiicola* | China | EU822319 | *Navy bean* | [43] |
| *C. cassiicola* | Cuba | HE605034 | *Carica papaya* | [39] |
| *C. cassiicola* | China | GQ381292 | *Momordica charantia* | [27] |
| *Corynespora cassiicola* | México | GU461301 | *Cucumis sativus* | [19] |
| *C. cassiicola* | USA (Georgia) | JQ717069 | *Gossypium* sp | [18] |
| *C. constrictum* | New Zeeland | JQ005238 | *Citrus limon* | [13] |
| *C. gloesporiodes* | India | HQ264178 | *Capsicum annuum* | [48] |
| *C. gloesporiodes* | India | JF710548 | *Mangifera indica* L | [11] |
| *C. gloesporiodes* | Brazil | JQ753973 | *Phaseolus. vulgaris* L | [24] |
| *C. parsonsiae* | New Zeeland | JQ005233 | *Parsonia capsularis* | [13] |
| *C. petchii* | Italy | JQ005223 | *Dracaena marginata* | [13] |
| *D. longicolla* | Costa Rica | KU204662 | *Protium costaricense* | [44] |
| *D. melonis* | Puerto Rico | KT972129 | *Crescentia portoricensis* | [35] |
| *D. sojae* | USA | KM279929 | *Glycine max* (L.) Merr | [15] |
| *Diaporthe* sp. | Panamá | EF423550 | *Protium costaricense* | [22] |
| *Diaporthe* sp. | Panamá | FJ799939 | *Cordia alliodora* | [56] |
| *D. stewartii* | Costa Rica | KU204429 | *Stylogyne laevis* | [44] |
| *Diplodia alatafructa* | South Africa | FJ888461 | *Pterocarpus angolensis* | [33] |
| *D. mutila* | Ventura, CA (USA) | JQ659283 | *Citrus limon* (variety Lisbon lemon) | [1] |
| *Diaporthe seriata* | China | HQ730975 | *Malus sylvestris* | [60] |
| *D. seriata* | Apulia, Italy | FJ481595 | *Vitis vinífera* | [9] |
| *Fusarium chlamydosporum* | China | HQ654898 | Unknown | [8] |
| *F. chlamydosporum* | Ghana | FJ545394 | *Theobroma cacao* | [2] |
| *F. equiseti* | Japan | AB425996 | *Triticum spp* | [12] |
| *F. equiseti* | Japan | AB272123 | *Triticum spp* | [12] |
| *F. equiseti* | Spain | FN394681 | *Holcus lanatus* | [46] |
| *F. incarnatum* | China | EU111657 | Aegiceras corniculatum | [28] |
| *F. incarnatum* | China | EF158029 | Wetland | [37] |
| *F. chlamydosporum* | China | JQ690082 | Melon plant | [61] |
| *F. equiseti* | India | HQ995668 | *Oriza sativa* | [6] |
| *F. incarnatum* | China | AB369420 | *Cotinus coggygria Scop* | [53] |
| *Glomerella cingulata* | India | GU810509 | Unknown | [42] |
| *G. cingulata* | Taiwan | FJ172236 | *Glycine max* | [10] |
| *G. cingulata* | India | JQ818200 | *Moringa oleifera* | [23] |
| *L. theobromae* | Texas (USA) | FJ790837 | *Vitis vinífera* | [55] |
| *L. theobromae* | USA | GU251121 | *Prunus dulcis,* | [25] |
| *L. theobromae* | Australia | GU199386 | *Andasonia digitata* | [45] |
| *L. theobromae* | Taiwán | GQ502455 | *Mangifera indica* | [36] |
| *L. theobromae* | Spain | EU600925 | *Vitis vinífera* | [32] |
| *L. theobromae* | China | GU134887 | Unknown | [29] |
| *L. theobromae* | Brazil | JF766989 | *Piper hispidum* | [38] |
| *L. theobromae* | Malaysia | HM466953 | *Jatropa curcas* | [51] |
| *L. theobromae* | China | EF641303 | *Taxus chinensis* | [34] |
| *L. theobromae* | USA | GU251121 | *Pistacia vera* | [25] |
| *L. theobromae* | Australia | GU199386 | *Andasonia digitata* | [45] |
| *Macrophomina phaseolina* | India | JQ954868 | Legumes (unknown) | [21] |
| *M. phaseolina* | China | JQ676193 | *Camptotheca cuminata,* | [50] |
| *M. phaseolina* | Spain | AM410964 | *Fragaria x ananassa* | [3] |
| *M. phaseolina* | China | HQ660594 | Mung bean *(Vigna radiata)* | [59] |
| *M. phaseolina* | USA (Arizona) | GU046891 | *Fraxinus* sp | [31] |
| *Penicillium citrinum* | México | EU833213 | Leaf litter | [17] |
| *P. citrinum* | Australia | KU059850 | *Nicotiana simulans* | [14] |
| *P. citrinum* | Brazil | KM613148 | Soil | [4] |
| *Penicillium* sp. | Argentina | KP195167 | *Ilex paraguariensis* | [40] |
| *Penicillium* sp. | Brazil | KC181929 | Wild shrub | [7] |
| *Phaeobotryon mamane* | Hawai (USA) | EU673336 | Unknown | [41] |
| *Phomopsis* sp. | Ecuador | HQ157158 | *Spondias mombin* | [5] |
| *R. bataticola*  *(M. phaseolina)* | India | JQ954878 | Legumes (unknown) | [21] |
| *Rhizoctonia bataticola*  *(M. phaseolina)* | India | HQ392815 | *Cicer arietinum*. | [47] |

# References

1. Adesemoye AO, Mayorquin JS, Wang DH, et al. Identification of species of Botryosphaeriaceae causing bot gummosis in citrus in California. Plant Dis. 2014; 98: 56-61.
2. Adu-Acheampong R, Archer S. Diversity fungi associated with Mirid (Hemiptera: *Miridae*) lesions feeding and dieback disease. Int J Agric Res. 2011; 6(9): 660-672.
3. Avilés M, Castillo, Bascon J, Zea-Bonilla, Martín-Sánchez PM, Pérez-Jiménez RM. First report of *Macrophomina phaseolina* causing crown and root rot of strawberry in Spain. Plant Pathol. 2008; 57: 382.
4. Barbosa RN, Pereira JDB, Oller CPM, Correia de Lima-Júnior N, et al. Aspergillus and *Penicillium* (Eurotiales: *Trichocomaceae*) in soils of the Brazilian tropical dry forest: diversity in an area of environmental preservation. Rev Biol Trop (Int J Trop Biol). 2016; 64(1): 45-53.
5. Bascom-Slack CA, Boulanger LA, Griffin MA, Spakowicz DJ, Nunez VP, Baraban EG, Berwick SA, Darnell AM, Eyler LE, Gallagher BD, Goldstein DW, Jefferson GKS, Jiang GY, Lazzarini TA, Martin KO, Maust MR, Pan L, Åortero CE, Rakhmankulova M, Szigety KM, Thakral D, Strobel SA. Comparison of endophytes isolated from rainforest plants. Unpublished. 2010.
6. Bashyal, B. M., & Aggarwal, R. (2013). Molecular identification of Fusarium spp. associated with bakanae disease of rice in India. Indian Journal of Agricultural Sciences,
7. Bashyal BM, Aggarwal R. Molecular identification of *Fusarium* species associated with bakanae disease of rice (*Oryza sativa*) in India. Indian J Agr Sci. 2013; 83: 72–77.
8. Bemiro MS. CO_2_ enrichment influences the spectral signature, development endophyte community in a tropical wild shrub. Unpublished. 2012.
9. Cao C, Song R. Direct submission. Unpublished. 2010.
10. Carlucci A, Lops F, Raimondo WL, Gentile V, et al. The *Botryosphaeria* species from vineyards of Apulia. Phytopathologia Mediteranea. 2009; 48: 180.
11. Chen LS, Liu CD, et al. PCR-based detection and differentiation of anthracnose pathogens, *Colletotrichum gloesporioides* and *C. truncatum*, from vegetable soybean in Taiwan. J. Phytopath. 2006; 154: 654-662.
12. Chowdappa P, Kumar MSP. Existence of two genetically distinct populations of *Colletotrichum gloeosporioides* Penz in mango from India. Pest Management in Horticultural Ecosystems. 2012; 18(2): 161-170.
13. Chung WH, Ishii H, Nishimura K, Oshima M, Iwama T, Yoshimatsu H. Genetic analysis and PCR-based identification of major Fusarium species causing head blight on wheat in Japan. J Gen Plant Pathol. 2008; 74: 364-374.
14. Damm U, Cannon PF, Woudenberg JHC, Johnston PR, et al. The *Colletotrichum boninense* species complex. Studies Mycol. 2012; 73: 1-36.
15. Dastogeer KMG, Wylie SJ, Li H, Sivasithamparam K. Association of endophytic mycobiota in native Asutralian Nicotiana spp.: Diversity, nice partitioning, growth character and their effect on host. Unpublished. 2015.
16. Divilov K, Walker DR. Taxonomy and biocontrol of *Diaporthe sojae* and screening for resistance to phomopsis seed decay caused by an atypical *Diaporthe sojae* isolate using various assays. M.Sc. Thesis, The University of Illions at Urbana-Champaign. 2014. <https://www.ideals.illinois.edu/bitstream/handle/2142/50652/Konstantin_Divilov.pdf?sequence=1>.
17. El-Hawary SS, Mohamed R, Abouzid SF, Rateb ME, Bakeer W. Potential fungal endophyte of *Solanum nigrum* L (Black nightshade) as novel source steroidal alkaloids and their glycosides. Unpublished. 2014.
18. Espino del Castillo A, Lappe P, Morales-Malacara JB, Marquez-Valdelamar L, Hernández M, García-Calderón N, Krasilnikov P. Diversity of microfungi of genus *Penicillium* and *Aspergillus* (Trichocomaceae) and yeast in soil from Los Riscos cave, Jalpan, Queretaro, Mexico. Unpublished. 2008.
19. Fulmer AM, Walls JT, Dutta B, Parkunan V, et al. First report of target spot caused by *Corynespora cassiicola* on cotton in Georgia. Plant Dise. 2012; 96(7): 1066.
20. García CL, Perez-Brito D, Tapia-Tussel R, et al. Corynespora leaf spot: A new disease on *Cucumis sativus* in Yucatan, Mexico. Unpublished. 2010.
21. Garzón-Grajales N, Almeciga-Díaz CJ, Robles-Camargo J. Characterization of isolated fungi from Guasca and Cruz verde wastelands (Cundimarca-Colombia). Unpublished. 2013.
22. Gautam R, Singh SK, Sharma V. Molecular diversity in root rot pathogen of arid legumes. Unpublished. 2012.
23. Gilbert GS, Webb CO. Phylogenetic signal in plant pathogen-host range. [Proc Natl Acad Sci U S A.](https://www.ncbi.nlm.nih.gov/pubmed/17360396) 2007; 104(12): 4979-83
24. GokulRaj K, Sundaresan N, Jagan EG, Rajapriya P, Muthumary J, Sridhar J, Pandi M. Phylogenetic reconstruction of endophytic fungal isolates using internal transcribed spacer 2 (ITS2) region. Bioinformation. 2014; 10(6): 320-328.
25. Gonzaga LL, Costa LEO, Queiroz MV. Endophytic fungi from the genus *Colletotrichum* are abundant in the *Phaseolus vulgaris* and have high genetic diversity. J Appl Microbiol. 2015; 118:485-496.
26. Inderbitzin P, Bostock RM, Michailides TJ. A six locus phylogeny reveals high species diversity in *Botryosphaeriaceae* from California almond. Mycologia. 2010; 102(6): 1350-1368.
27. Kaul RK, Joshi P. Nanoparticle-producing fungi isolated arid soils of India. Unpublished. 2012.
28. Li BJ, Zhao YJ, Gao W, Shi YX, Xie XW. First report of target leaf spot caused by *Corynespora cassi*icola on Balsam Pear in China. Plant Dis. 2010; 94: 127.
29. Li YL, Ding Yi, Groth I, Menzel KD, Peschel G, et al. Pyrrole and indole alkaloids from an endophytic *Fusarium incarnatum* (HKI00504) isolated from the mangrove plant *Aegiceras corniculatum*. J Asian Natural Products Res. 2008; 10(8): 765-770.
30. Liu N, Tian L. Isolation of microorganisms from plants habitats and study of isolation methods. Unpublished. 2009.
31. Mahmodi F, Kadir J, Puteh A, Mui Yun W, Nasehi A. Characterization and pathogenicity of *Colletotrichum capsici* isolated from chilli (*Capsicum* spp) and vegetables crops in Malaysia. Unpublished. 2012.
32. Manici L, Caputo F, Accinelli C. Spatial and temporal genetic variability of *Macrophomin*a populations from mid latitudes. Unpublished. 2009.
33. Martin MT, Martin L, de Francisco MT, Cobos R. First report of *Lasidiplodia theobromae* and *Crytovalsa ampelina* associated with grapevine decline from Castilla y León, Spain. Plant Dis. 2009; 93(5): 545-545.
34. Mehl JWM, Slippers B, Roux J, Wingfield MJ. *Botryosphaeriaceae* associated with *Pterocarpus angolensis* (kiaat) in South Africa. Mycologia. 2011; 103(3): 534-553.
35. Miao Z, Wang Y, Yu X, Guo B, Tang K. A new endophytic taxane production fungus from *Taxus chinensis*. Appl Biochem Microbiol. 2009; 45: 51-86.
36. Monclova-Santana C, Rivera FM, Rivera-Vargas LL. Pathogenic threats of ten endangered plant species of the karst region of Puerto Rico. Unpublished. 2015.
37. Ni HF, Liou RF, Hung TH, et al. First report of fruit rot disease of Mango caused by *Botryosphaeria dothidea* and *Neofusicoccum mangiferae* in Taiwan. Plant Dis. 2010; 94(1): 128-128.
38. Nie M, Luo J, Xiao M, Chen J, Bao K, Zhang W, Chen J, Li B. Structural differences between *Fusarium* strains investigated by FT-IR spectroscopy. Biochemistry (Moscow). 2007; 72(1): 61-67.
39. Orlandelli RC, Alberto RN, Almeida TT, et al. In vitro antibacterial activity of crude extracts produced by endophytic fungi isolated from *Piper hispidum* Sw. J Appl Pharmaceutical Sci. 2014; 2(10): 137-141.
40. Peňa-Marey M, Banguela CA, Ramos-González PL, Castaňeda R, Hernández L. Molecular variability of *Corynespora cassiicola* isolate from Cuba. Unpublished. 2010.
41. Pérez ML, Collavino MM, Sansberro PA, Mroginski LA, Galdeano E. Diversity of endophytic fungal and bacterial communities in *Ilex paraguariensis* grown under field conditions. World J Microbiol Biotechnol. 2016. doi:10.1007/s11274-016-2016-5
42. Phillips AJL, Lopes J, Abdollahzadeh J, et al. Resolving the *Diplodia* complex on apple and other *Rosaceae* hosts. Persoonia. 2012; 29: 29-38.
43. Prema RT, Prabakar K, Raguchander T, Karthikeyan G, Ramjegathesh R. Molecular characterization of *Colletotrichum musae* through RAPD analysis. Unpublished. 2010.
44. Qi Y, Xie Y, Pu J, Zhang X. Identification of *Corynespora cassiico*la isolated from navy bean. Unpublished. 2008.
45. Rojas JK, Murillo C, Clardy J, Tamayo G. Fungal and bioactive compounds from tropical forests of Costa Rica. Unpublished. 2015.
46. Sakalidis ML, Hardy GE, Burgess TI. Endophytes as potential pathogens of the baobab species *Adansonia gregorii*: a focus on the Botryosphaeriaceae. Fungal Ecol. 2011; 4: 1-14.
47. Sánchez SM, Bills GF, Acuña LD, Zabalgogeazcoa I. Endophytic mycobiota of leaves and roots of the grass Holcus lanatus. Fungal Diversity. 2010; 41: 115-123.
48. Sharma M, Ghosh R, Tilak RS, Pandei S. Intra population diversity in *Rhizoctonia bataticola* causing dry root rot of chickpea (*Cicer arietinum* L.) in India. African J Microbiol Res. 2012; 6(37): 6653-6660.
49. Sharma PN, Sharma P, Sharma S, Katoch A, Banya K, Nag R. Characterization of *Colletotrichum* species associated with fruit rot of chilli (*Capsicum annuum*). Unpublished. 2010.
50. Srinivas C, Udayashankar AC, Nayaka SC. Chitosan induced resistance to anthracnose in chilli caused by *Colletotrichum capsici*. Unpublished. 2011.
51. Su H, kang JC, Cao JJ, Mo L, D.Hyde K. Medicinal plant endophytes produce analogous bioactive compounds. Chiang Mai J. Sci. 2014; 41(1): 1-13.
52. Sulaiman R, Thanarajoo SS. First reort of *Lasiodiplodia theobromae* causing stem canker of *Jatropha curcas* in Malaysia. Plant Dis. 2012; 96(2): 767.
53. The LY, Latiffah Z. Occurrence and molecular characterization of *Aspergillus* species in beach sand. Unpublished. 2012.
54. Tian CM. ITS region of fungi. Unpublished. 2007.
55. Torres-Calzada C, Tapia-Tussel R, Higuera-ciapara I, Perez-Brito D. Morphological, pathological and genetic diversity of *Colletotrichum* species responsible for anthracnose in papaya (*Carica papaya* L). Eur J Plant Pathol. 2013; 135: 67-79.
56. Ùrbez-Torres JR, Adams P, Kamas J, Gubler WD. Identification, incidence, and pathogenicity of fungal species associated with grapevine dieback in Texas. Am J Enol Vitic. 2009; 60: 497-507.
57. Van Bael SA, Fernández-Marín H, Valencia MC, Rojas EI, et al. Two fungal symbioses collide: endophytic fungi are not welcome in leaf-cutting ant gardens. Proc Biol Sci. 2009; 276(1666): 2419-2426.
58. Yadav M, Yadav A, Kumar S, Parkash JY. Spatial and seasonal influences on culturable endophytic mycobiota associated with different tissues of *Eugenia jambola* Lam. and their antibacterial activity against MDR strains. BMC Microbiol. 2016. doi: 10.1186/s12866-016-0664-0
59. Zhang H. Direct submission. Unpublished. 2011.
60. Zhang JQ, Zhu ZD, Duan CX, Wang XM, Li HJ. First report of charcoal rot caused by *Macrophomina phaseolina* on Mung bean in China. Plant Dis. 2011; 95(7): 872.
61. Zhao N, Huang LL, Wang WW. Identification of apple ring rot isolates in Shaanxi provinces based on ITS sequences of rDNA. Unpublished. 2010.
62. Zhao J. Isolation and identification of fungal isolates caused melon plant diseases, unpublished. 2012.
63. Zhou S, Stanosz GR. Relation among *Botryosphaeria* species and associated anomorphic fungi inferred from the analyses of ITS and 5.8S rDNA sequences. Mycologia. 2001; 93(3): 516-527.
