# Supplementary figures and images for: Seedborne Pathogenic Fungi in Common Bean (Phaseolus vulgaris cv. INTA Rojo) in Nicaragua
Source: PLoS One. 2016 Dec 20;11(12):e0168662. doi: 10.1371/journal.pone.0168662 (PMC5173241; doi:10.1371/journal.pone.0168662)

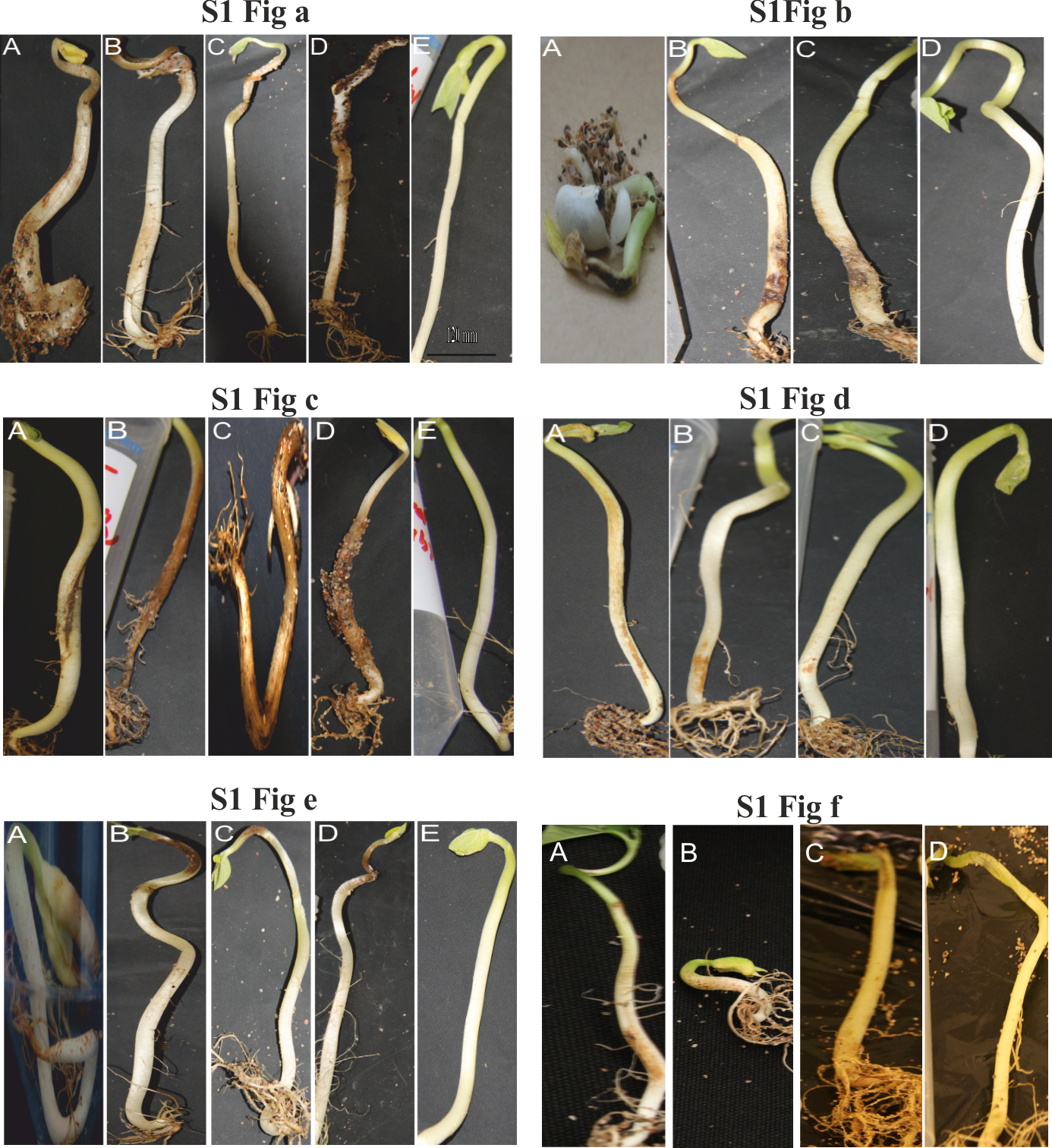

Supplement: S1 Fig — Photographs were taken at 20 dpi. Panel (a) Fusarium spp., (b) Macrophomina phaseolina, (c) Lasiodiplodia theobromae, (d) Penicillium citrinum, (e) Colletotrichum capsici (photos A and B) and Colletotrichum gloeosporioides (photos C and D), and (f) Aspergillus flavus. The right-most photograph in each panel shows the mock-inoculated control (photograph E in panels a, c and e, and photograph D in panels b, d and f). (TIF) [file pone.0168662.s002.tif]
